# Prioritizing Electrocardiogram Interpretation for Emergency Medicine Residency Training: A Modified Delphi Study

**DOI:** 10.1002/aet2.70158

**Published:** 2026-04-29

**Authors:** Jennifer Yee, Geremiha Emerson, Richard V. Thompson, Kelsey H. Jordan, Sorabh Khandelwal

**Affiliations:** ^1^ Department of Emergency Medicine The Ohio State University College of Medicine Columbus Ohio USA; ^2^ Office of Curriculum and Scholarship The Ohio State University College of Medicine Columbus Ohio USA

**Keywords:** cardiology, clinical competency, clinical skills, electrocardiograms, emergency medicine, medical education graduate, standards

## Abstract

**Background:**

Rapid, accurate interpretation of electrocardiogram (ECG) patterns is a key skill for emergency medicine (EM) physicians, but there is no published consensus on which ECG patterns should be prioritized. Within Competency‐Based Medical Education (CBME) frameworks, diagnostic skills such as ECG interpretation require articulated performance expectations and assessment targets. Our study objective was to identify the most important ECG patterns for CBME alignment based on the dual constructions of “importance to identify” and “clinical significance”.

**Methods:**

A three‐round electronic modified Delphi survey was constructed for a convenience sample of geographically‐diverse EM fellows and faculty across the United States (*N* = 14). Panelists were also asked to rate patterns based on importance to identify and perceived clinical significance using Likert scales.

**Results:**

Fourteen EM attending physicians completed the modified Delphi. Most panelists had 6–10 years of clinical experience (43%), practiced within the East North Central region (45%), and were affiliated with university‐based programs (91%). A list of 78 ECG patterns was generated. The most important to identify and clinically significant ECG patterns included rhythms covered by Advanced Cardiac Life Support teaching and patterns indicative of acute coronary syndrome. Several patterns (e.g., atrial fibrillation) were rated as “most important to identify” but lower in clinical significance, suggesting some patterns are foundational for diagnostic reasoning but may not independently mandate immediate intervention without appropriate clinical context. Conversely, cardiac glycoside toxicity demonstrated high clinical significance despite lower importance‐to‐identify ratings, highlighting conditions in which management urgency is high but diagnosis relies more on clinical context than ECG pattern recognition alone.

**Conclusions:**

Through a modified Delphi study, we generated a list of ECG patterns based on importance to identify and perceived clinical significance. Educators may use this list to guide ECG curriculum development, create assessment strategies, and align with educational targets.

## 1 Introduction

Electrocardiograms (ECGs) are a key diagnostic tool to assess for acute coronary syndromes, to evaluate for underlying causes of syncope or dizziness, and a screening tool for certain electrolyte abnormalities or toxidromes. Rapid and accurate interpretation of ECG patterns is a key skill for emergency medicine (EM) physicians, particularly for patients who may have a time‐sensitive pathology such as an acute occlusive myocardial infarction. There is an exhaustive list of ECG patterns caused by electrical, structural, and/or secondary pathologies. It can be overwhelming for EM trainees to determine which ECG patterns they need to prioritize learning for their clinical practice, both in terms of importance due to high‐risk underlying pathology or clinical significance, which would subsequently change management strategies.

There is a foundational shift in graduate medical education to adopt Competency‐Based Medical Education (CBME) principles to prepare trainees to achieve desired health and health care outcomes [1]. This includes an outcomes‐based approach to the design, implementation, assessment, and evaluation of physicians and physician training programs [2, 3]. Within CBME frameworks, diagnostic skills such as ECG interpretation require clearly articulated performance expectations and assessment targets [4]. Given time and resource constraints in residency training, residency curriculum leaders require consensus on which ECG patterns warrant instructional priority and assessment. Importantly, the educational importance of teaching an ECG pattern may not align perfectly with its immediate clinical significance. Conflating these constructs risks both curricular inefficiency and assessment misalignment. Drawing on established frameworks in health professions education, importance to identify reflects a mastery‐oriented learning target–the expectation that a trainee independently recognizes a pattern as part of developing foundational diagnostic reasoning, regardless of clinical context. This construct aligns most directly with the *knows how* and *shows how* levels of Miller's pyramid [5] and informs decisions about curriculum sequencing, learning objectives, and formative assessment. Clinical significance, by contrast, reflects the urgency and magnitude of management consequences if a pattern is missed, and is most relevant to patient safety‐driven prioritization, high‐stakes summative assessment design, and entrustment decisions within CBME frameworks [6, 7].

**FIGURE 1:**
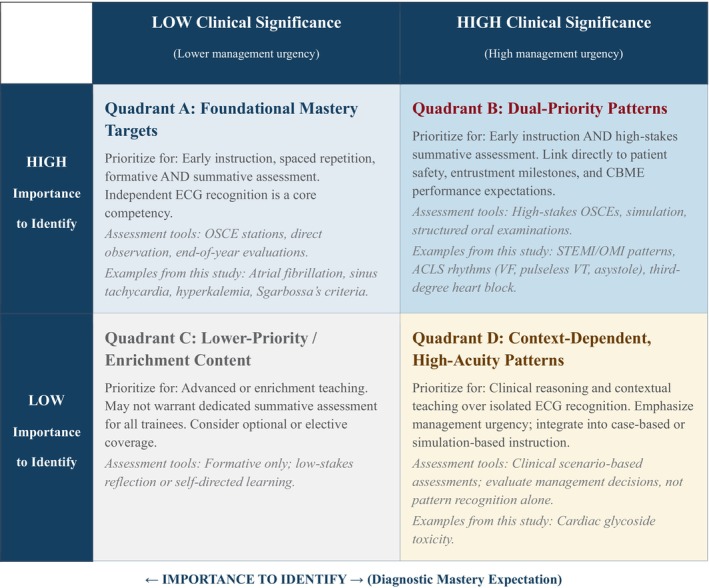
Conceptual framework for applying 'importance to identify' and 'clinical significance' in ECG curriculum design and assessment.

A pattern may warrant early mastery instruction because it is diagnostically foundational (high importance to identify) without independently mandating immediate intervention in every clinical context (lower clinical significance). Conversely, a pattern may carry high management urgency when present (high clinical significance) yet be sufficiently rare or context‐dependent that independent ECG recognition is not considered a universal competency target. Explicitly separating these constructs allows educators to build curriculum sequences and assessment blueprints that are simultaneously competency‐aligned and responsive to patient safety priorities (Figure 1).

Resources for learning ECG patterns exist for EM residents (e.g., *Foundations of Emergency Medicine* [8], EM:RAP [9]), but there is no published consensus on which ECG patterns should be prioritized for EM residents. Two previous studies on which ECG patterns should be taught to EM residents were limited in terms of geographical location (with only one specific area surveyed) [10], types of residency programs included (only including academic‐based settings) [11], and roles of responding panelists [11].

Educators require clear performance expectations and assessment targets within the CBME framework, including for ECG education for EM resident training. We have conducted a geographically‐ and panelist‐diverse modified Delphi via electronic survey to inform EM educators and residency leadership which ECG patterns should be prioritized during EM residency training. Therefore, the objective of this study was to identify ECG patterns to teach EM residents based on perceived importance to identify and clinical significance using a modified Delphi approach.

## 2 Methods

### 2.1 Study Design

We selected a modified Delphi approach to allow structured expert consensus [12] in adherence to DELPHISTAR guidelines [13, 14] while incorporating an initial investigator‐generated ECG list informed by existing curricula and prior literature. The Delphi process was designed to: (1) generate a comprehensive list of ECG patterns relevant to EM residency training, (2) prioritize ECG patterns based on importance to identify, and (3) prioritize ECG patterns based on perceived “clinical significance,” reflecting the urgency and potential impact on emergency department management. Importance to identify was defined as the perceived necessity for a resident to independently recognize a pattern on ECG regardless of clinical context.

This study functions as clarification research, as outlined by Cook et al. [15], aiming to delineate which ECG patterns should be prioritized in EM training. It also contributes to the early steps of curriculum development described in Kern's model [16] by identifying and ranking educational needs through expert consensus. As recommended by Witkin and Altschuld [17], a small‐to‐medium group of 19 panelists was originally engaged and panelist contributions were kept anonymous. This study was reviewed and deemed exempt by our institutional IRB. No conflicts of interest were declared. The study received no external funding. We did not use any large language models or artificial intelligence‐generated content tools to develop any portion of this manuscript.

### 2.2 Panelist Recruitment

Our research team consisted of faculty and staff from our Department of Emergency Medicine and the College of Medicine educational research teams. This group used convenience sampling to recruit geographically diverse EM fellows and faculty from across the United States (US) (Table 1). We intentionally targeted individuals with active roles in resident education, curriculum design, or ECG instruction to ensure content and educational expertise. The research question—which ECG patterns should be prioritized in EM residency training–is fundamentally a curricular question, distinct from asking which patterns are most clinically important in general practice. Accordingly, the relevant expertise is that of EM medical educators rather than specialists from other disciplines. Cardiologists and trainees were therefore intentionally excluded: cardiologists set clinical standards that extend beyond EM scope of practice, and trainees, while important stakeholders, lack the educational expertise to define curricular priorities. This sampling strategy reflects a principled, scope‐specific design rather than a limitation of convenience. Contacts were encouraged to recruit any additional fellows or faculty at their institution who were involved with resident ECG education. A panel size of 10–15 participants was selected a priori based on Delphi methodology literature [17], suggesting that small expert panels are appropriate when participant expertise is high and the domain is well‐defined. Humphrey‐Murto et al.12 similarly affirm that smaller Delphi panels are defensible when participants share a well‐defined, homogeneous area of expertise—a criterion met by our panel of EM educators with direct responsibility for resident ECG instruction.

**TABLE 1:** Modified delphi panelist's demographic details for electrocardiogram (ECG) pattern identification and prioritization for EM residency training study.

ID	Institution name	Geographic area based on AMA's FREIDA	Residency program category	Years of clinical practice (post‐residency)	Current roles	Do you deliver the majority of ECG educational content to your EM residents, or is this split between individuals?
1	The Ohio State University	East North Central	University‐based	6–10 years	Assistant or associate program director	Yes
2	The Ohio State University	East North Central	University‐based	21+ years	Program director	No
3	University of Cincinnati	East North Central	University‐based	6–10 years	Assistant or associate program director	Yes
4	University of Cincinnati	East North Central	University‐based	6–10 years	Assistant or associate program director	Equal distribution across two or more faculty/fellows
5	University of Michigan	East North Central	University‐based	< 1–5 years	Medical education fellow	No
6	University of Michigan	East North Central	University‐based	< 1–5 years	Medical education fellow	No
7	Northwestern University	East North Central	University‐based	6–10 years	Assistant or associate program director	Equal distribution across two or more faculty/fellows
8	University of Vermont	New England	University‐based	< 1–5 years	Assistant or associate program director	No
9	University of Vermont	New England	University‐based	< 1–5 years	Core faculty member	Yes
10	Louisiana State University Health Sciences Center New Orleans	West South Central	Community‐based, university‐affiliated	6–10 years	Assistant or associate program director	No
11	University of California, Davis	Pacific	University‐based	6–10 years	Assistant or associate program director	Yes
12	Oregon Health and Science University	Pacific	University‐based	11–15 years	Core faculty member	Equal distribution across two or more faculty/fellows
13	Stanford University	Pacific	University‐based	21+ years	Core faculty member	Yes
14	University of Texas Southwestern	West South Central	University‐based	11–15 years	Program director	No

Abbreviations: AMA, American medical association; ECG, electrocardiogram; EM, emergency medicine. FREIDA, fellowship and residency electronic interactive database; ID, identification.

### 2.3 Development of the Initial ECG List

Before round one, three EM faculty in the original research team (the first, second, and senior authors) created a list of ECG patterns based on Foundations [8], Penalo et al. [10], Patocka, Turner, and Wiseman [11], and their personal expertise. The list was subsequently categorized by underlying pathologies. This pre‐populated list was intended to provide a structured starting point while allowing panelists to suggest additional ECG patterns and refinements.

### 2.4 Delphi Rounds

The research staff from the College of Medicine created and disseminated an electronic survey (Qualtrics International Inc., Provo, Utah, USA) to disseminate three rounds of our modified Delphi [18]. To maximize our response rate, reminders for completion were disseminated on days 2,8, and 14 for each round [19].

### 2.5 Round 1

Panelists were provided the list of generated ECG patterns and were asked to assign each pattern a score on a 5‐point Likert scale: level of importance to be taught during EM residency training, with 1 representing not at all important and 5 as ‘extremely important’. The panel was also asked to submit additional ECG patterns or free‐text comments. Demographic information (i.e., current educational position, years in clinical practice after residency training, and who delivers the majority of ECG educational content to their residents) was requested at the end of the round 1 survey. Program type (e.g., “university‐based”) and different US geographical region designations mirrored those used by the American Medical Association's (AMA) Fellowship and Residency Electronic Interactive Database (FREIDA) [20].

### 2.6 Round 2

Two ECG patterns suggested through round 1's free‐text responses were added: atrial fibrillation with an accessory pathway (Wolff‐Parkinson‐White) and bidirectional ventricular tachycardia. Panelists were shown the mean importance scores from round 1 and asked to rate each ECG pattern using two separate 4‐point Likert‐type scales: (scale 1) “importance to identify” (1 = does not need to identify; 4 = must be able to identify) and (scale 2) “clinical significance” (1 = would not affect EM management; 4 = must immediately act upon to limit morbidity and mortality). Panelists were also provided with a free‐text comment box to suggest additional patterns and clarify concerns about wording or categorization.

### 2.7 Round 3

Minor edits were made to ECG descriptions for clarity based on round 2 feedback, e.g., changing “pulmonary embolism findings” to “in a patient with suspected pulmonary embolism”. Panelists were shown mean scores from round 2 and asked to re‐score each ECG pattern using the same two scales. Panelists were again provided an optional free‐text comment field to identify any remaining concerns or missed patterns.

Three rounds were conducted to allow initial exploration, refinement, and stabilization of responses, consistent with recommended Delphi methodology [17].

### 2.8 Data Analysis

As appropriate at each round, means were calculated for importance to identify and clinical significance scores for each ECG pattern. Although Likert‐type data are ordinal, we used mean scores for consistency with prior Delphi studies and to enable rank‐ordering [21]. As a technique recommended by Altschuld and Thomas [22] and performed by Mitzman et al. [23], we calculated a “strength score” by multiplying each Likert rating value by the frequency of that rating and summing across ratings, providing a measure that accounts for both rating magnitude and response distribution. Strength scores for all 78 ECG patterns are reported in Supplemental Tables 1 and 2 and were used to inform rank ordering alongside mean scores. We also calculated “percent endorsed,” defined as the proportion of panelists selecting the highest category (i.e., must be able to identify or must immediately act upon). Consensus was operationally defined as ≥ 80% endorsement of the highest category, with unanimous (100%) endorsement representing strong consensus.

## 3 Results

A convenience sample of 14 EM physicians from multiple US regions served as panelists. (Table 1) Ten panelists completed all three rounds, and one additional panelist completed rounds 2 and 3 only. One panelist did not complete the round 3 “clinical significance” ratings. Attrition after round 1 primarily occurred among participants with fewer years of post‐residency clinical experience and included both panelists from New England and two panelists from the East North Central region. Our panelist in a community‐based, university‐affiliated program completed all three rounds. (Figure S1).

The 14 panelists who completed round 1 provided demographics. Half of respondents were assistant or associate program directors (*n* = 7, 50%) and slightly fewer (*n* = 6, 43%) had 6–10 years of clinical practice after residency training. Five panelists (36%) primarily perform education delivery, 6 (43%) responded that another faculty member or fellow primarily provides resident ECG education, and 3 (21%) responded that there is equal residency ECG education distribution across two or more faculty and/or fellows. The greatest proportion of panelists were from the East North Central region (45%) and predominantly affiliated with university‐based residency programs (91%). (Table 1).

For importance to identify (Table 2), 13 items out of 78 (16.7%) were given the highest score and were endorsed by all panelists. Four of these were occlusive myocardial infarctions and one was Sgarbossa's criteria [24]. The other patterns are covered by Advanced Cardiac Life Support teaching [25] other than the pattern suggestive of hyperkalemia.

**TABLE 2:** Top 13 Electrocardiogram (ECG) patterns from importance to identify scores.

Category	Rhythm	Mean	SD	Strength	% Endorsed
Tachydysrhythmias	Atrial fibrillation	4.0	0.0	44	100%
	Sinus tachycardia	4.0	0.0	44	100%
	Ventricular tachycardia	4.0	0.0	44	100%
Bradycardias & AV Blocks	Second‐degree, Mobitz type 2	4.0	0.0	44	100%
	Third‐degree/complete heart block	4.0	0.0	44	100%
Pulseless rhythms	Asystole	4.0	0.0	44	100%
	Ventricular fibrillation	4.0	0.0	44	100%
Electrolyte abnormalities	Hyperkalemia	4.0	0.0	44	100%
ACS/OMI/ACS equivalents	Sgarbossa criteria (LBBB/paced rhythms)	4.0	0.0	44	100%
	STEMI (anterior)	4.0	0.0	44	100%
	STEMI (inferior)	4.0	0.0	44	100%
	STEMI (lateral)	4.0	0.0	44	100%
	STEMI (posterior)	4.0	0.0	44	100%

Abbreviations: ACS, acute coronary syndrome; AV, atrioventricular; LBBB, left bundle branch block; OMI, occlusive myocardial infarction; SD, standard deviation.; STEMI, ST‐elevation myocardial infarction.

For clinical significance (Table 3), 6 ECG patterns (7.7%) were given the highest score and were endorsed by all panelists. Four of these were patterns of occlusive myocardial infarctions, while two (asystole and third‐degree heart block) are covered by Advanced Cardiac Life Support teaching [25].

**TABLE 3:** Top 10 Electrocardiogram (ECG) patterns from clinical significance scores.

Category	Rhythm	Mean	SD	Strength	% Endorsed
Bradycardias & AV Blocks	Third‐degree/complete heart block	4.0	0.0	40	100%
Pulseless rhythms	Asystole	4.0	0.0	40	100%
ACS/OMI/ACS equivalents	STEMI (anterior)	4.0	0.0	40	100%
	STEMI (inferior)	4.0	0.0	40	100%
	STEMI (lateral)	4.0	0.0	40	100%
	STEMI (right‐sided)	4.0	0.0	40	100%
	STEMI (posterior)	3.9	0.32	39	90%
Tachydysrhythmias	Ventricular tachycardia	3.9	0.32	39	90%
Toxicology/Environmental	Cardiac glycoside toxicity	3.9	0.320	39	90%
Additional ECGs added	Bidirectional ventricular tachycardia	3.8	0.63	38	90%

Abbreviations: ACS, acute coronary syndrome; AV, atrioventricular; ECG, electrocardiogram; LBBB, left bundle branch block; OMI, occlusive myocardial infarction; SD, standard deviation; STEMI, ST‐elevation myocardial infarction.

Two patterns were given the highest score and endorsement for importance to identify but not clinical significance: atrial fibrillation and sinus tachycardia. None of the large discrepancies had a higher score for clinical significance than importance to identify. (Table 4) These discordant ratings are noteworthy: atrial fibrillation, for example, achieved unanimous endorsement for importance to identify but did not reach the same threshold for clinical significance, suggesting panelists view it as foundational for diagnostic reasoning rather than independently mandating immediate intervention. Conversely, cardiac glycoside toxicity received lower importance to identify ratings but relatively higher clinical significance ratings, reflecting a pattern where management urgency is high but diagnosis is driven more by clinical context than ECG pattern recognition alone.

**TABLE 4:** Electrocardiogram (ECG) patterns demonstrating largest discrepancies between Importance to Identify and clinical significance scores.

Category	Rhythm	% (Imp)	% (Clin)	Mean (Imp)	Mean (Clin)	SD (Imp)	SD (Clin)
Pericarditis	Diffuse ST elevation	81.82	0.0	3.82	2.9	0.4	0.32
Acute ischemia without meeting OMI criteria	ST depressions	90.91	10.0	3.91	2.9	0.3	0.57
Bradycardias & AV blocks	Sinus bradycardia	90.91	20.0	3.91	2.5	0.3	0.85
Tachydysrhythmias	Atrial fibrillation	100.0	30.0	4.0	3.3	0.0	0.48
Tachydysrhythmias	Sinus tachycardia	100.0	33.33	4.0	2.89	0.0	0.93
Pericarditis	Diffuse PR depression	63.64	0.0	3.55	2.8	0.69	0.42
Syncope	Long QTc	90.91	30.0	3.91	3.1	0.3	0.74
Bradycardias & AV blocks	2:1 block	80.0	20	3.8	2.8	0.42	0.92
Hypertrophy	Left ventricular hypertrophy	54.55	0.0	3.36	2.0	0.81	0.47
Bradycardias & AV blocks	Second‐degree, Mobitz type 1	81.82	30.0	3.82	2.6	0.4	1.17
Bradycardias & AV blocks	High‐grade AV block (> 2:1 conduction)	81.82	30.0	3.73	3.1	0.65	0.74
Ectopy	Premature ventricular contractions (PVCs)	45.45	0.0	3.36	1.7	0.67	0.48

Abbreviations: AV, atrioventricular; Clin, clinical significance; Imp, importance to identify; SD, standard deviation; STEMI, ST‐elevation myocardial infarction; OMI, occlusive myocardial infarction.

The complete list of ECG pattern ratings is available in Tables S1 and S2.

## 4 Discussion

This modified Delphi study generated a prioritized list of ECG patterns for EM residency training while distinguishing between importance to identify and perceived clinical significance. This distinction provides a nuanced framework for curricular prioritization, assessment planning, and alignment with CBME principles.

The patterns rated both most important to identify and most clinically significant included rhythms covered by Advanced Cardiac Life Support teaching [25] and patterns indicative of occlusive myocardial infarction. We suspect this is because patients with these patterns are most likely to be critically ill and require urgent management. Several ECG patterns were rated as important to identify but lower in clinical significance, including atrial fibrillation, sinus tachycardia, hyperkalemia, and patterns meeting Sgarbossa's criteria [24]. These findings suggest that some ECG patterns are foundational for diagnostic reasoning and longitudinal competence but may not independently mandate immediate intervention without appropriate clinical context. Conversely, cardiac glycoside toxicity demonstrated high clinical significance despite lower importance‐to‐identify ratings, highlighting conditions in which management urgency is high but diagnosis relies more heavily on clinical context than ECG pattern recognition alone. To operationalize this distinction for educational practice, we propose a conceptual two‐dimensional framework organizing ECG patterns across these constructs (Figure 1), which may serve as a practical tool for curriculum sequencing and assessment blueprinting in EM residency programs.

When synthesized through an educational lens, our findings reveal three distinct pattern categories that warrant different curricular approaches. First, *immediate action‐triggering patterns* (e.g., ventricular fibrillation, pulseless ventricular tachycardia, occlusive myocardial infarctions) scored highest on both constructs, representing patterns where recognition directly precipitates time‐sensitive interventions. These patterns should dominate early post‐graduate year (PGY)‐1 instruction and be assessed through high‐fidelity simulation and objective structured clinical examinations, as failure to identify these patterns risks immediate patient harm. Second, *foundational diagnostic reasoning patterns* (e.g., atrial fibrillation, sinus tachycardia) demonstrated high importance‐to‐identify but lower clinical significance scores, reflecting patterns that are frequently encountered and diagnostically essential yet rarely trigger immediate management changes without clinical contextualization. For these patterns, educators should emphasize longitudinal competence through spaced repetition, workplace‐based assessment, and integration with clinical reasoning frameworks rather than isolated high‐stakes testing. Third, *context‐dependent high‐acuity patterns* (e.g., cardiac glycoside toxicity) exhibited high clinical significance despite lower recognition priority, suggesting conditions in which management urgency is paramount but ECG interpretation is only one component of a broader diagnostic synthesis. For these patterns, case‐based teaching that emphasizes clinical presentation, medication history, and laboratory findings, rather than pattern memorization alone, may be most pedagogically efficient. This tripartite framework allows program directors to allocate limited instructional time proportional to both learning objectives and patient safety imperatives.

Two previous studies created prioritized EM ECG lists. Penalo et al.'s aim was to create a general importance ranking for EM and critical care nursing and multidisciplinary audiences rather than a specific focus on EM resident education [10]. Their panel had a minority of EM physicians and was geographically limited. Furthermore, only the top 20 ECG patterns were listed per discipline (nursing, cardiology, and EM). Patocka, Turner, and Wiseman's panel only included Canadian EM program directors [11]. Similar to our list, most of their generated high‐ranking patterns included ACLS‐taught rhythms. However, neither group examined a ranking of perceived clinical significance of ECG patterns. Separating these constructs may help educators differentiate ECG patterns that warrant early mastery and summative assessment from those better addressed through advanced or context‐specific teaching. These constructs are not perfectly orthogonal, as management urgency is partly context‐dependent; ratings reflect perceived rather than empirically validated relationships between ECG pattern recognition and patient management.

The American Board of Emergency Medicine (ABEM) Model of the Clinical Practice of Emergency Medicine [26] outlines required content for residency training; however, time and educational resources during EM training are limited. Although ECG interpretation is listed as a required diagnostic and therapeutic skill, the model does not specify which ECG patterns should be prioritized. In accordance with programmatic assessment principles, clearly defined educational priorities allow educators to focus teaching and assessment efforts. Our goal was to create a consensus‐based framework identifying ECG patterns based on importance to identify and perceived clinical significance. Critically, the construct of importance to identify is inherently scope‐of‐practice dependent: what an EM resident must independently recognize differs meaningfully from what a cardiology fellow or internal medicine trainee must master. This makes EM educator‐specific consensus the methodologically appropriate starting point for this line of inquiry and frames the absence of cardiologist or trainee panelists as a scoping decision rather than an oversight.

These consensus ratings can be used to support curriculum sequencing and assessment blueprinting in EM residency programs. Patterns rated high in both importance to identify and clinical significance may be prioritized for early instruction, deliberate practice, and summative assessment (e.g., ECG stations in objective structured clinical examinations, structured oral examinations, or high‐stakes end‐of‐year evaluations). In contrast, patterns rated high in importance but lower in clinical significance may be emphasized longitudinally through spaced exposure and formative assessment, reflecting the need for clinical context rather than immediate action. Programs may also use these ratings to align teaching and assessment with local milestones, entrustment decisions, and programmatic assessment strategies. For example, a residency program might structure its PGY‐1 ECG curriculum to prioritize mastery of ACLS rhythms and occlusive myocardial infarction recognition through weekly simulation sessions with competency‐based progression, while introducing atrial fibrillation and bundle branch blocks through longitudinal case conferences that emphasize diagnostic reasoning rather than isolated pattern recognition. Assessment blueprints might allocate 60% of ECG testing weight to dual‐priority patterns (Quadrant B in Figure 1), 30% to foundational reasoning patterns (Quadrant A), and 10% to context‐dependent patterns (Quadrant D), with lower‐priority patterns (Quadrant C) reserved for advanced or elective teaching. Similarly, entrustment decisions for independent ECG interpretation might require demonstrated competence in Quadrant B patterns before PGY‐2 advancement, while competence in Quadrant A patterns could be assessed longitudinally across PGY‐2 and PGY‐3 years through workplace‐based assessments and portfolio review. Such structured prioritization addresses the tension between comprehensive content coverage and finite educational time, ensuring that assessment efforts are calibrated to both learning objectives and patient safety imperatives.

Importantly, this prioritization framework remains relevant in an era of increasing artificial intelligence (AI)‐assisted ECG interpretation. Although AI tools show promise in pattern detection [27], physician competence remains essential: algorithms require clinical contextualization, may fail during system downtime or in resource‐limited settings, and cannot independently synthesize ECG findings with medication history, electrolyte abnormalities, or clinical trajectory. The distinction between importance to identify and “clinical significance” may in fact become more useful as educators determine which patterns require independent mastery versus AI‐augmented interpretation.

## 5 Limitations

Generalizability may be most limited by lack of panelist diversity, particularly for clinical practice settings. Most panelists were affiliated with university‐based residency programs and certain geographic regions were overrepresented. This sampling may bias results toward ECG patterns emphasized in academic or geographic‐specific curricula and may underrepresent priorities in community‐based training environments. Panel attrition occurred across rounds and may have influenced consensus thresholds. Notably, panelists who did not complete all rounds tended to have fewer years of clinical experience. Importantly, with only 10 panelists completing all three rounds, the 80% consensus threshold required agreement from just eight individuals. This makes the consensus threshold more susceptible to individual variation and should be considered when interpreting results. We did not conduct sensitivity analyses using alternate consensus thresholds, which is a limitation. As noted above, these exclusions of trainee and cardiologist panelists were intentional: the research question targets EM curricular prioritization, for which EM educator judgment is the primary relevant expertise. Future work should examine whether trainee self‐assessments of ECG learning needs and cardiologist perspectives on clinical importance yield concordant or divergent priorities, which would further validate or refine this framework.

Lastly, the modified Delphi process has intrinsic potential disadvantages. Panelist responses represent expert judgment; high levels of agreement do not necessarily ensure content or response validity. We sought to minimize impact of group dynamics by allowing panelists to individually add ECG patterns or to raise questions about proposed items [28]. Therefore, interpretations of study findings should take these limitations into account, pending additional research.

## 6 Conclusion

This modified Delphi study identified ECG patterns prioritized by EM educators based on both importance to identify and perceived clinical significance. Patterns that scored highly in both categories included rhythms covered by Advanced Cardiac Life Support teaching [25], hyperkalemia, and patterns indicative of acute coronary syndrome and occlusive myocardial infarction. These findings provide a practical framework to guide ECG curriculum development, assessment strategies, and CBME‐aligned educational planning in EM residency training. Future work should seek to validate these priorities through prospective mapping to resident milestone assessments, OSCE blueprinting, and entrustable professional activity alignment. Broader panelist samples–including community‐based educators, trainees, and cardiology specialists–would strengthen the generalizability of subsequent consensus efforts. Adoption of this framework by residency programs may also enable comparative research on ECG education outcomes across diverse training settings.

## Author Contributions

Study concept and design: Jennifer Yee, Geremiha Emerson, Richard V. Thompson, Kelsey H. Jordan, Sorabh Khandelwal. Acquisition of the data: Jennifer Yee, Geremiha Emerson, Richard V. Thompson, Kelsey H. Jordan, Sorabh Khandelwal. Analysis and interpretation of the data: Jennifer Yee, Geremiha Emerson, Richard V. Thompson, Kelsey H. Jordan, Sorabh Khandelwal. Drafting of the manuscript: Jennifer Yee, Geremiha Emerson, Richard V. Thompson, Kelsey H. Jordan, Sorabh Khandelwal. Critical revision of the manuscript for important intellectual content: Jennifer Yee, Geremiha Emerson, Richard V. Thompson, Kelsey H. Jordan, Sorabh Khandelwal. Statistical expertise: Richard V. Thompson, Kelsey H. Jordan. Acquisition of funding: n/a.

## Funding

The authors have nothing to report.

## Conflicts of Interest

The authors declare no conflicts of interest.

## Supporting information


**Supplementary Figure S1:** Panelist Participation.

**Supplementary Table S1:** Supplement 1: Importance to Identify (Full List).

**Supplementary Table S2:** Supplement 2: Clinical Significance (Full List).

## Data Availability

The data that supports the findings of this study are available in the Supporting Information of this article.
