# Supplementary figures and images for: Prioritizing Electrocardiogram Interpretation for Emergency Medicine Residency Training: A Modified Delphi Study
Source: AEM Educ Train. 2026 Apr 29;10(2):e70158. doi: 10.1002/aet2.70158 (PMC13128520; doi:10.1002/aet2.70158)

Figure 1: Panelist Participation

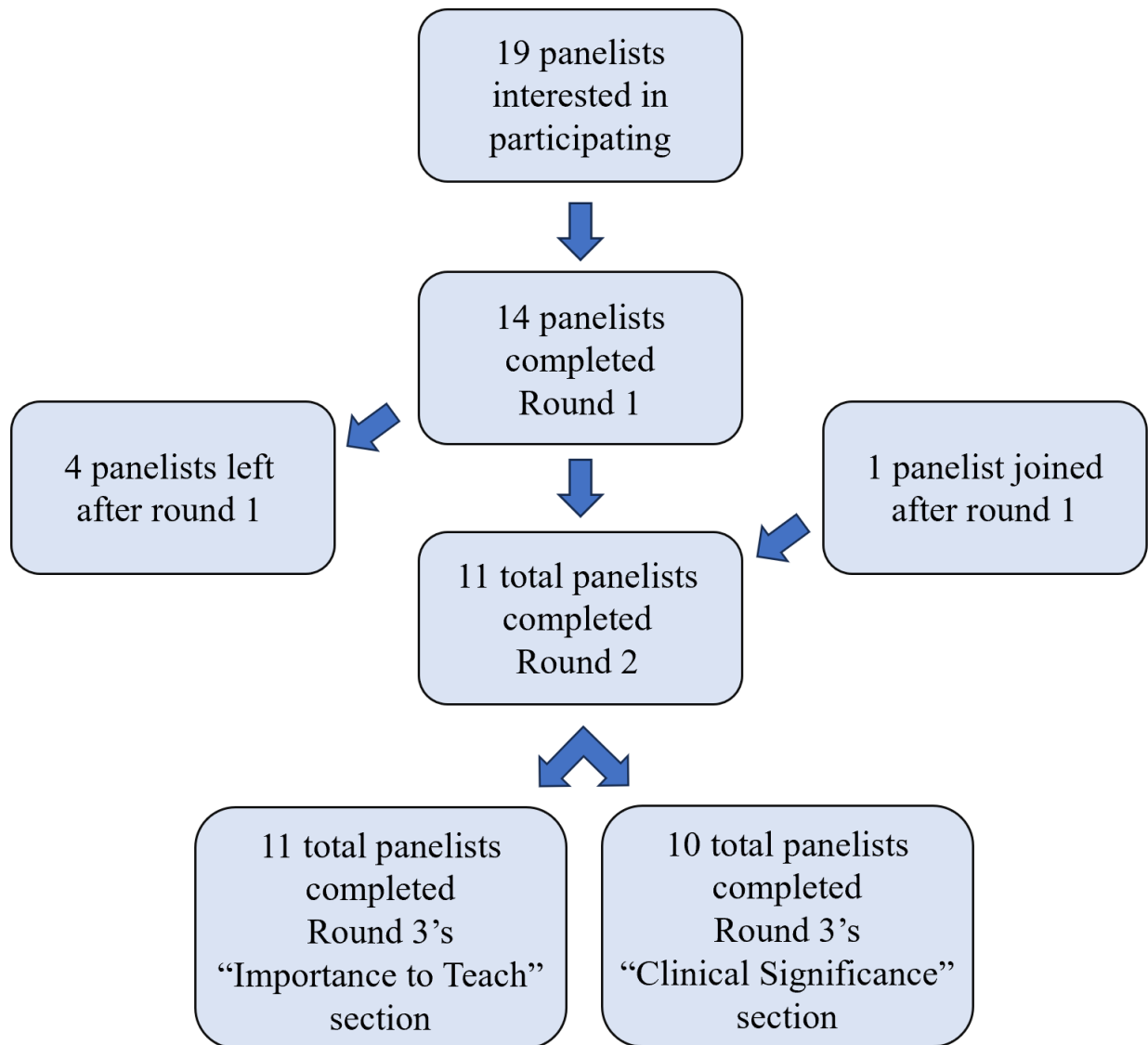

Supplement: Supplementary file 1 — Supplementary Figure S1: Panelist Participation. [file AET2-10-e70158-s001.pdf]
