# Supplementary material for: Prioritizing Electrocardiogram Interpretation for Emergency Medicine Residency Training: A Modified Delphi Study
Source: AEM Educ Train. 2026 Apr 29;10(2):e70158. doi: 10.1002/aet2.70158 (PMC13128520; doi:10.1002/aet2.70158)
Supplement: Supplementary file 2 — Supplementary Table S1: Supplement 1: Importance to Identify (Full List). [file AET2-10-e70158-s002.docx]

Supplement 1: Importance to Identify (Full List)

| **Category** | **Rhythm** | **Mean** | **SD** | **%**  **Endorsed** | **Strength** |
| --- | --- | --- | --- | --- | --- |
| Tachydysrhythmias | Sinus tachycardia | 4.00 | 0.00 | 100% | 44 |
|  | Atrial fibrillation | 4.00 | 0.00 | 100% | 44 |
| Bradycardias and AV blocks | Second-degree,  Mobitz type 2 | 4.00 | 0.00 | 100% | 44 |
| Acute coronary syndrome (ACS) / Occlusive myocardial infarction (OMI) / Acute Coronary Syndrome (ACS) equivalents | Sgarbossa criteria  (for LBBB/paced rhythms) | 4.00 | 0.00 | 100% | 44 |
| Tachydysrhythmias | Ventricular tachycardia | 4.00 | 0.00 | 100% | 44 |
| Electrolyte abnormalities | Hyperkalemia | 4.00 | 0.00 | 100% | 44 |
| Bradycardias and AV blocks | Third-degree/  complete heart block | 4.00 | 0.00 | 100% | 44 |
| Pulseless rhythms | Asystole | 4.00 | 0.00 | 100% | 44 |
|  | Ventricular fibrillation | 4.00 | 0.00 | 100% | 44 |
| Acute coronary syndrome (ACS) / Occlusive myocardial infarction (OMI) / Acute Coronary Syndrome (ACS) equivalents | STEMI (anterior) | 4.00 | 0.00 | 100% | 44 |
|  | STEMI (inferior) | 4.00 | 0.00 | 100% | 44 |
|  | STEMI (lateral) | 4.00 | 0.00 | 100% | 44 |
|  | STEMI (posterior) | 4.00 | 0.00 | 100% | 44 |
| Findings indicating acute ischemia without meeting STEMI/OMI criteria | ST depressions | 3.91 | 0.30 | 91% | 43 |
| Bradycardias and AV blocks | Sinus bradycardia | 3.91 | 0.30 | 91% | 43 |
| Syncope | Long QTc | 3.91 | 0.30 | 91% | 43 |
| Acute coronary syndrome (ACS) / Occlusive myocardial infarction (OMI) / Acute Coronary Syndrome (ACS) equivalents | STEMI (right-sided) | 3.91 | 0.30 | 91% | 43 |
| Pericarditis | Diffuse ST elevation | 3.82 | 0.40 | 82% | 42 |
| Bradycardias and AV blocks | Second-degree, Mobitz type 1 | 3.82 | 0.40 | 82% | 42 |
| Tachydysrhythmias | Atrial fibrillation with accessory pathway (WPW) | 3.82 | 0.40 | 82% | 42 |
| Bradycardias and AV blocks | 2:1 block | 3.80 | 0.42 | 80% | 38 |
| ST-elevation differential | Brugada | 3.73 | 0.47 | 73% | 41 |
| Bradycardias and AV blocks | High-grade AV block (>2:1 conduction pattern) | 3.73 | 0.65 | 82% | 41 |
| Toxicology and Environmental | Sodium channel blocker toxicity | 3.73 | 0.47 | 73% | 41 |
| Tachydysrhythmias | Atrial flutter | 3.64 | 0.50 | 64% | 40 |
|  | Ventricular tachycardia (bidirectional) | 3.64 | 0.67 | 73% | 40 |
| Acute coronary syndrome (ACS) / Occlusive myocardial infarction (OMI) / Acute Coronary Syndrome (ACS) equivalents | deWinter's T-waves | 3.64 | 0.50 | 64% | 40 |
| Toxicology and Environmental | Osborne waves | 3.64 | 0.50 | 64% | 40 |
| Pericarditis | Diffuse PR depression | 3.55 | 0.69 | 64% | 39 |
| In a patient with a suspected pulmonary embolism | RBBB | 3.55 | 0.52 | 55% | 39 |
| Acute coronary syndrome (ACS) / Occlusive myocardial infarction (OMI) / Acute Coronary Syndrome (ACS) equivalents | aVR ST-elevation with diffuse depressions | 3.55 | 0.69 | 64% | 39 |
|  | Wellen's T-waves with delayed R-wave progression | 3.55 | 0.52 | 55% | 39 |
| Bradycardias and AV blocks | First-degree AV block | 3.45 | 0.82 | 64% | 38 |
| Toxicology and Environmental | Cardiac glycoside toxicity | 3.45 | 0.52 | 45% | 38 |
| Pacemaker dysfunction | Failure to sense | 3.45 | 0.52 | 45% | 38 |
| Electrolyte abnormalities | Hypokalemia | 3.45 | 0.69 | 55% | 38 |
| Hypertrophy | Left ventricular hypertrophy (LVH) | 3.36 | 0.81 | 55% | 37 |
| Ectopy | Premature ventricular contractions (PVCs) | 3.36 | 0.67 | 45% | 37 |
| Toxicology and Environmental | Beta-blocker/Calcium channel blocker toxicity | 3.36 | 0.50 | 36% | 37 |
| ST-elevation differential | Early repolarization | 3.36 | 0.50 | 36% | 37 |
| Pacemaker dysfunction | Oversensing | 3.36 | 0.67 | 45% | 37 |
|  | Undersensing | 3.36 | 0.67 | 45% | 37 |
| In a patient with a suspected pulmonary embolism | ST-elevation in aVR | 3.27 | 0.47 | 27% | 36 |
| Findings indicating underlying CAD without acute ischemia | Wellen's warning | 3.27 | 0.90 | 45% | 36 |
| Acute coronary syndrome (ACS) / Occlusive myocardial infarction (OMI) / Acute Coronary Syndrome (ACS) equivalents | Bifascicular block: LBBB | 3.18 | 0.87 | 45% | 35 |
| In a patient with a suspected pulmonary embolism | S1Q3T3 | 3.18 | 0.60 | 27% | 35 |
| Tachydysrhythmias | Atrioventricular nodal reentrant tachycardia (AVnRT) | 3.18 | 0.75 | 36% | 35 |
|  | Atrioventricular reentrant tachycardia (AVRT) | 3.18 | 0.75 | 36% | 35 |
| In a patient with a suspected pulmonary embolism | Anterior T-wave inversions | 3.09 | 0.70 | 27% | 34 |
| Electrolyte abnormalities - | Hypocalcemia | 3.09 | 0.54 | 18% | 34 |
| Pericarditis | Absence of ST depressions in all leads but aVR | 3.00 | 1.00 | 36% | 33 |
| Acute coronary syndrome (ACS) / Occlusive myocardial infarction (OMI) / Acute Coronary Syndrome (ACS) equivalents | Bifascicular block: right bundle branch block (RBBB) and left anterior fascicular block (LAFB) | 3.00 | 0.77 | 27% | 33 |
|  | Bifascicular block: RBBB + left posterior fascicular block (LPFB) | 3.00 | 0.77 | 27% | 33 |
| ST-elevation differential | Pre-excitation | 3.00 | 0.89 | 27% | 33 |
| Syncope | Short QTc | 3.00 | 1.10 | 36% | 33 |
| Hypertrophy | Right ventricular hypertrophy (RVH) | 3.00 | 0.63 | 18% | 33 |
| Syncope | Early repolarization | 3.00 | 0.77 | 27% | 33 |
| Pacemaker dysfunction | Pacemaker-mediated tachycardia | 3.00 | 1.10 | 36% | 33 |
| Electrolyte abnormalities | Hypercalcemia | 3.00 | 0.63 | 18% | 33 |
| Pericarditis | ST depression and PR elevation in aVR | 2.91 | 0.94 | 27% | 32 |
| Findings indicating underlying CAD without acute ischemia | LV aneurysm pattern | 2.91 | 0.83 | 18% | 32 |
| Syncope | Catecholaminergic polymorphic ventricular tachycardia | 2.82 | 1.08 | 36% | 31 |
|  | Arrhythmogenic right ventricular cardiomyopathy (ARVC) | 2.82 | 0.98 | 27% | 31 |
| Acute coronary syndrome (ACS) / Occlusive myocardial infarction (OMI) / Acute Coronary Syndrome (ACS) equivalents | Barcelona criteria [for left bundle branch block (LBBB)/paced rhythms] | 2.82 | 0.87 | 27% | 31 |
| Findings indicating acute ischemia without meeting STEMI/OMI criteria | aVL T-wave inversions | 2.82 | 0.87 | 18% | 31 |
| Ectopy | Premature atrial contractions (PACs) | 2.73 | 0.79 | 9% | 30 |
| Findings indicating acute ischemia without meeting STEMI/OMI criteria | Pseudonormalization of T-waves | 2.73 | 0.79 | 9% | 30 |
| Electrolyte abnormalities | Hypomagnesemia | 2.64 | 1.03 | 18% | 29 |
| Tachydysrhythmias | Junctional tachycardia | 2.64 | 0.81 | 9% | 29 |
|  | Atrial tachycardia | 2.55 | 0.69 | 9% | 28 |
|  | Multifocal atrial tachycardia (MAT) | 2.55 | 0.82 | 9% | 28 |
| Ectopy | Premature junctional complexes (PJCs) | 2.55 | 0.69 | 0% | 28 |
| Pericarditis | Downsloping TP segments (Spodick sign) | 2.45 | 0.82 | 9% | 27 |
| Tachydysrhythmias | Sinus node re-entrant tachycardia | 2.36 | 0.92 | 9% | 26 |
| Bradycardias and AV blocks | Sinus node exit block | 2.36 | 0.81 | 9% | 26 |
| Findings indicating underlying CAD without acute ischemia | Regional QRS fragmentation | 2.27 | 0.65 | 0% | 25 |
| Tachydysrhythmias | Paroxysmal atrial tachycardia | 1.90 | 0.74 | 0% | 19 |
| Hypertrophy | Atrial hypertrophy | 1.82 | 0.75 | 0% | 20 |

Abbreviations:

Standard Deviation (SD)

Atrioventricular (AV)

Acute Coronary Syndrome (ACS)

Occlusive Myocardial Infarction (OMI)

Left Bundle Branch Block (LBBB)

ST-Elevation Myocardial Infarction (STEMI)

Electrocardiogram (ECG)
