# Supplementary material for: Prioritizing Electrocardiogram Interpretation for Emergency Medicine Residency Training: A Modified Delphi Study
Source: AEM Educ Train. 2026 Apr 29;10(2):e70158. doi: 10.1002/aet2.70158 (PMC13128520; doi:10.1002/aet2.70158)
Supplement: Supplementary file 3 — Supplementary Table S2: Supplement 2: Clinical Significance (Full List). [file AET2-10-e70158-s003.docx]

Supplement 2: Clinical Significance (Full List)

| **Category** | **Rhythm** | **Mean** | **SD** | **% Endorsed** | **Strength** |
| --- | --- | --- | --- | --- | --- |
| Tachydysrhythmias | Ventricular tachycardia (bidirectional) | 4.00 | 0.00 | 100% | 40 |
| Bradycardias and AV blocks | Third-degree/  complete heart block | 4.00 | 0.00 | 100% | 40 |
| Pulseless rhythms | Asystole | 4.00 | 0.00 | 100% | 40 |
|  | Ventricular fibrillation | 4.00 | 0.00 | 100% | 40 |
| Acute coronary syndrome (ACS) / Occlusive myocardial infarction (OMI) / Acute Coronary Syndrome (ACS) equivalents | STEMI (anterior) | 4.00 | 0.00 | 100% | 40 |
|  | STEMI (inferior) | 4.00 | 0.00 | 100% | 40 |
|  | STEMI (lateral) | 4.00 | 0.00 | 100% | 40 |
|  | STEMI (posterior) | 4.00 | 0.00 | 100% | 40 |
| Toxicology and Environmental | Cardiac glycoside toxicity | 3.90 | 0.32 | 90% | 39 |
|  | Sodium channel blocker toxicity | 3.90 | 0.32 | 90% | 39 |
| Tachydysrhythmias | Ventricular tachycardia | 3.90 | 0.32 | 90% | 39 |
| Electrolyte abnormalities | Hyperkalemia | 3.90 | 0.32 | 90% | 39 |
| Acute coronary syndrome (ACS) / Occlusive myocardial infarction (OMI) / Acute Coronary Syndrome (ACS) equivalents | STEMI (right-sided) | 3.90 | 0.32 | 90% | 39 |
| Toxicology and Environmental | Beta-blocker/  Calcium channel blocker toxicity | 3.80 | 0.42 | 80% | 38 |
| Acute coronary syndrome (ACS) / Occlusive myocardial infarction (OMI) / Acute Coronary Syndrome (ACS) equivalents | Sgarbossa criteria (for LBBB/paced rhythms) | 3.80 | 0.42 | 80% | 38 |
| Tachydysrhythmias | Atrial fibrillation with accessory pathway (WPW) | 3.80 | 0.42 | 80% | 38 |
| Bradycardias and AV blocks | Second-degree, Mobitz type 2 | 3.70 | 0.48 | 70% | 37 |
| Acute coronary syndrome (ACS) / Occlusive myocardial infarction (OMI) / Acute Coronary Syndrome (ACS) equivalents | Wellen's T-waves with delayed R-wave progression | 3.70 | 0.67 | 80% | 37 |
|  | deWinter's T-waves | 3.70 | 0.48 | 70% | 37 |
| Toxicology and Environmental | Osborne waves | 3.60 | 0.52 | 60% | 36 |
| Syncope | Catecholaminergic polymorphic ventricular tachycardia | 3.50 | 0.71 | 60% | 35 |
| Electrolyte abnormalities | Hypokalemia | 3.40 | 0.52 | 40% | 34 |
| Tachydysrhythmias | Atrial fibrillation | 3.30 | 0.48 | 30% | 33 |
| Bradycardias and AV blocks | High-grade AV block (>2:1 conduction pattern) | 3.30 | 0.67 | 40% | 33 |
| Pacemaker dysfunction | Pacemaker-mediated tachycardia | 3.30 | 0.48 | 30% | 33 |
| Tachydysrhythmias | Atrioventricular nodal reentrant tachycardia (AVnRT) | 3.30 | 0.67 | 40% | 33 |
|  | Atrioventricular reentrant tachycardia (AVRT) | 3.30 | 0.67 | 40% | 33 |
| ST-elevation differential | Brugada | 3.20 | 0.63 | 30% | 32 |
| Acute coronary syndrome (ACS) / Occlusive myocardial infarction (OMI) / Acute Coronary Syndrome (ACS) equivalents | aVR ST-elevation with diffuse depressions | 3.20 | 0.63 | 30% | 32 |
| Pacemaker dysfunction | Failure to sense | 3.20 | 0.42 | 20% | 32 |
|  | Oversensing | 3.20 | 0.42 | 20% | 32 |
|  | Undersensing | 3.20 | 0.42 | 20% | 32 |
| Syncope | Long QTc | 3.10 | 0.74 | 30% | 31 |
| Tachydysrhythmias | Atrial flutter | 3.10 | 0.88 | 30% | 31 |
| Acute coronary syndrome (ACS) / Occlusive myocardial infarction (OMI) / Acute Coronary Syndrome (ACS) equivalents | Barcelona criteria [for left bundle branch block (LBBB)/paced rhythms] | 3.10 | 0.74 | 30% | 31 |
| Bradycardias and AV blocks | 2:1 block | 3.00 | 0.67 | 20% | 30 |
| Syncope | Arrhythmogenic right ventricular cardiomyopathy (ARVC) | 3.00 | 0.67 | 20% | 30 |
| Electrolyte abnormalities | Hypercalcemia | 3.00 | 0.67 | 20% | 30 |
| Pericarditis | Diffuse ST elevation | 2.90 | 0.32 | 0% | 29 |
| Findings indicating acute ischemia without meeting STEMI/OMI criteria | ST depressions | 2.90 | 0.57 | 10% | 29 |
| Findings indicating underlying CAD without acute ischemia | Wellen's warning | 2.90 | 0.74 | 20% | 29 |
| Findings indicating acute ischemia without meeting STEMI/OMI criteria | aVL T-wave inversions | 2.90 | 0.74 | 20% | 29 |
| Pericarditis | Diffuse PR depression | 2.80 | 0.42 | 0% | 28 |
| Tachydysrhythmias | Sinus node re-entrant tachycardia | 2.80 | 0.92 | 30% | 28 |
| Electrolyte abnormalities | Hypomagnesemia | 2.80 | 0.42 | 0% | 28 |
| Tachydysrhythmias | Junctional tachycardia | 2.80 | 0.63 | 10% | 28 |
| Findings indicating acute ischemia without meeting STEMI/OMI criteria | Pseudonormalization of T-waves | 2.80 | 0.63 | 10% | 28 |
| Electrolyte abnormalities | Hypocalcemia | 2.70 | 0.48 | 0% | 27 |
| Tachydysrhythmias | Sinus tachycardia | 2.67 | 0.87 | 22% | 24 |
| In a patient with a suspected pulmonary embolism | ST-elevation in aVR | 2.60 | 0.52 | 0% | 26 |
| In a patient with a suspected pulmonary embolism | Anterior T-wave inversions | 2.60 | 0.52 | 0% | 26 |
| Pericarditis | ST depression and PR elevation in aVR | 2.60 | 0.52 | 0% | 26 |
| Bradycardias and AV blocks | Sinus node exit block | 2.60 | 0.84 | 20% | 26 |
| Tachydysrhythmias | Atrial tachycardia | 2.60 | 0.84 | 20% | 26 |
| Bradycardias and AV blocks | Sinus bradycardia | 2.50 | 0.85 | 20% | 25 |
| In a patient with a suspected pulmonary embolism | RBBB | 2.50 | 0.53 | 0% | 25 |
| Pericarditis | Absence of ST depressions in all leads but aVR | 2.50 | 0.71 | 0% | 25 |
| Syncope | Short QTc | 2.50 | 0.85 | 10% | 25 |
| In a patient with a suspected pulmonary embolism | S1Q3T3 | 2.50 | 0.71 | 10% | 25 |
| Pericarditis | Downsloping TP segments (Spodick sign) | 2.50 | 0.53 | 0% | 25 |
| Bradycardias and AV blocks | Second-degree, Mobitz type 1 | 2.40 | 1.07 | 20% | 24 |
| Findings indicating underlying CAD without acute ischemia | LV aneurysm pattern | 2.40 | 0.52 | 0% | 24 |
| Acute coronary syndrome (ACS) / Occlusive myocardial infarction (OMI) / Acute Coronary Syndrome (ACS) equivalents | ABifascicular block: LBBB | 2.33 | 0.71 | 11% | 21 |
| Tachydysrhythmias | Multifocal atrial tachycardia (MAT) | 2.30 | 0.67 | 10% | 23 |
| Findings indicating underlying CAD without acute ischemia | Regional QRS fragmentation | 2.30 | 0.48 | 0% | 23 |
| Acute coronary syndrome (ACS) / Occlusive myocardial infarction (OMI) / Acute Coronary Syndrome (ACS) equivalents | Bifascicular block: right bundle branch block (RBBB) and left anterior fascicular block (LAFB) | 2.20 | 0.42 | 0% | 22 |
|  | RBBB + left posterior fascicular block (LPFB) | 2.20 | 0.42 | 0% | 22 |
| ST-elevation differential | Pre-excitation | 2.20 | 0.42 | 0% | 22 |
| Tachydysrhythmias | Paroxysmal atrial tachycardia | 2.20 | 1.03 | 20% | 22 |
| Hypertrophy | Left ventricular hypertrophy (LVH) | 2.10 | 0.32 | 0% | 21 |
|  | Right ventricular hypertrophy (RVH) | 2.10 | 0.57 | 0% | 21 |
| Syncope | Early repolarization | 2.00 | 0.82 | 10% | 20 |
| Bradycardias and AV blocks | First-degree AV block | 1.90 | 0.99 | 10% | 19 |
| ST-elevation differential | Early repolarization | 1.90 | 0.32 | 0% | 19 |
| Ectopy | Premature ventricular contractions (PVCs) | 1.80 | 0.42 | 0% | 18 |
|  | Premature junctional complexes (PJCs) | 1.70 | 0.67 | 0% | 17 |
|  | Premature atrial contractions (PACs) | 1.50 | 0.53 | 0% | 15 |
| Hypertrophy | Atrial hypertrophy | 1.40 | 0.52 | 0% | 14 |

Abbreviations:

Standard Deviation (SD)

Atrioventricular (AV)

Acute Coronary Syndrome (ACS)

Occlusive Myocardial Infarction (OMI)

Left Bundle Branch Block (LBBB)

ST-Elevation Myocardial Infarction (STEMI)

Electrocardiogram (ECG)
